# The lifeworld of people who ruminate: a qualitative phenomenological study

**DOI:** 10.3389/fpsyt.2026.1649971

**Published:** 2026-06-10

**Authors:** Aleš Oblak, Sara Rigler, Nika Kovačič, Liam Korošec Hudnik, Urban Kordeš, Jurij Bon, Borut Škodlar

**Affiliations:** 1 Laboratory for Cognitive Neuroscience and Psychopathology, University Psychiatric Clinic Ljubljana, Ljubljana, Slovenia; 2 Department of Psychology, Faculty of Arts, University of Ljubljana, Ljubljana, Slovenia; 3 Center for Cognitive Science, Faculty of Education, University of Ljubljana, Ljubljana, Slovenia; 4 Department of Psychiatry, Faculty of Medicine, University of Ljubljana, Ljubljana, Slovenia

**Keywords:** depression, lifeworld analysis, micro-phenomenology, phenomenological psychopathology, qualitative phenomenology, ruminating

## Abstract

**Background:**

Ruminations are persistent, repetitive, and often distressing thoughts centered on negative events, mood states, and psychiatric symptoms. Recognized as a maladaptive cognitive process, ruminating contributes to the onset and persistence of psychiatric disorders and is strongly linked to negative emotional states, self-criticism, and suicidality. Despite its clinical significance, a comprehensive, phenomenological understanding of ruminations remains lacking.

**Objectives:**

This study aims to provide a detailed, descriptive account of ruminations from the perspective of lifeworld analysis, focusing on embodiment, space, time, emotions, social relationships, and values. The goal is to enhance clinical understanding of the lived experience of ruminating and generate hypotheses for future research.

**Methods:**

A qualitative phenomenological approach was employed, combining micro-phenomenological interviews and lifeworld analysis. Data were collected from 51 participants, including both normative and clinical samples. A total of 107 interviews were conducted, focusing on 79 experiential episodes.

**Results:**

Ruminating is an epistemic practice, driven by a need to resolve uncertainty and create meaning. Ruminations are triggered by a collapse of commonsense understanding, leading to intellectualization of daily life and detachment from intuitive or embodied responses. Ruminative episodes are characterized by feelings of paralysis, emptiness, and problematic relationships with knowledge.

**Conclusion:**

Rather than being a maladaptive pattern of thinking, ruminating appears to constitute a complex lifeworld. This observation calls for reconceptualization of ruminations from a unified symptom towards a system of interrelated and extended altered experiences.

## 1 Introduction

Ruminations are ongoing, repetitive, and extended thoughts about negative events, one’s lowered mood, and psychiatric symptoms (1, 2). This pattern of thinking is recognized as a key maladaptive cognitive process that contributes to the development and persistence of psychiatric disorders (3). Unlike mental phenomena that are defined by their specific content (e.g., monothematic delusions), ruminations are characterized mainly by their perservative nature and their ability to trigger negative emotional states (2).

Tendency to ruminate has been shown to remain relatively stable even in light of significant increases in stress (4–8). Ruminating involves hard-to-control thoughts that patients find irritating and unnecessary (9). These thoughts usually lack clear conclusions and attempts to control them can increase anxiety (10). They often have a pseudophilosophical character (9). Ruminations appear to people as solution-focused. When ruminating, they believe that they are gaining deeper insight into their problems (11). Ruminations rarely lead to adaptive outcomes, as individuals focus more on their lack of motivation than on problem-solving. They are marked by negative self-criticism, low self-confidence, and a perceived lack of control (11, 12). They are strongly linked to both suicidal behavior and non-suicidal self-injury (13–19). Ruminative thinking is often triggered by loneliness (20). Research suggests that women are more prone to rumination than men (21), and this difference has been shown to partially explain why depression rates vary between genders (4, 21–23) However, this effect has not been consistently observed (24, 25). Recent findings further indicate that the relationship between rumination and depression may vary across cultural contexts (26).

Ruminations are a transdiagnostic symptom (27, 28). Recent meta-analytic and large-scale quantitative studies have consistently shown that ruminations are most strongly associated with depression and anxiety (29), while also extending to a broader spectrum of psychopathology. They occur in both internalizing and externalizing disorders (30). They tend to be made worse by engagement with social media (31). They are bidirectionally associated with sleep disorders (32, 33), which in and of themselves have an important role in the occurrence and maintenance of psychiatric disorders (34). Ruminating prolongs depressive episodes (8) and people who respond to stress with them have more frequent depressive episodes and a higher risk of a first-episode depression (35, 36). Recent findings further support the role of rumination in depression, showing that both trait and state ruminating predict depressive symptom severity, progression and treatment effectiveness, while also being associated with cognitive impairments such as reduced working memory capacity and disrupted reinforcement learning processes (37, 38). In addition to depression, ruminating is associated with anxiety throughout the lifespan (39, 40), including older adults, where it contributes to increased symptom severity (41). Repeated negative thinking has been shown to be the strongest psychological predictor of dementia in old age (42). Ruminating often occurs in eating disorders (43) as well as obsessive-compulsive disorder (44). It has also been observed in borderline personality disorder, where it is strongly connected to affective instability, unstable relationships, identity disturbance and impulsivity (45, 46). Ruminating occurs across a range of other conditions and contexts, including autism spectrum disorder (in the form of disorder-specific repetitive negative thinking (47)), climate anxiety (48) and in individuals exposed to adverse or stressful experiences in childhood, suggesting that it may represent a common maladaptive cognitive response across both clinical and non-clinical populations (49).

There are similarities between ruminating and other types of dysfunctional thinking, such as maladaptive daydreaming (50), psychotic hyper-reflexivity (51), anxiety sensitivity (i.e., the phenomenon wherein patients once having had a panic attack tend to worry about the recurrence of another one and therefore become vary of intense emotional states, including positive ones) (52, 53), and sticky thoughts (54, 55). Ruminations have a moderating function in the occurrence of referential delusions (56). However, the specific differences in the phenomenology and pathophysiology between these disparate phenomena are yet to be established. Behavioral (57) and neuroimaging studies (58) show that rumination is a symptom of depression that can occur independently of mood symptoms like dysphoria or anhedonia. Since ruminating is particularly strongly present in affective disorders (13, 59), the present investigation will focus primarily on depression and anxiety.

Ruminating has been a perennially discussed symptom of psychiatric disorders. In the 9^th^ century work *Maṣāliḥ al-Abdān wa al-Anfus*, which is considered to be a forerunner of cognitive behavioral therapy, ruminating is described as “whispers of the soul.” (60) The majority of the text is dedicated to dealing with ruminating. We can implicitly see the importance of ruminating already in Beck’s (61) cognitive theory of depression that focuses on negative self- and world-schemata. Martin and Tesser’s (62) account treats ruminating as a byproduct of how goal pursuit is regulated. When people perceive a discrepancy between their current state and an important but unresolved end goal, attention becomes repeatedly and involuntarily drawn back to it, producing persistent, recursive thinking about the discrepancy. Thus, ruminating is not random negative thinking but a kind of self-regulating negative feedback loop. (Perceived) feedback about insufficient progress keeps the representation of the goal actively at the forefront of one’s awareness, narrowing attention around the failure. Nolen-Hoeksema’s (8) response style theory places emphasis on dealing with one’s persistent negative feelings. On this view, ruminating is conceptualized as a maladaptive emotion regulation strategy. Similarly, Park’s (63, 64) meaning making-model understands ruminating after stressful events as an effort to reconcile it with one’s beliefs, goals, and sense of coherence about the world. Ruminating therefore amounts to a specific style of appraisal of emotionally salient events. Watkins’s (65) processing mode theory proposes that ruminating is maintained by an abstract, evaluative, why-focused cognitive style, and that shifting to a more concrete, specific, how-focused mode of thinking reduces its emotional impact. Watkins and Roberts’ (3) HExAGoN model conceptualizes ruminating as arising from the interaction of six maintaining processes (including abstract processing, negative bias, impaired problem-solving, unconstructive goal management, emotional processing deficits, and social factors), which together trap individuals in persistent, maladaptive repetitive thinking. Neuroscientific accounts emphasize dysconnectivity in the default mode network (58, 66). They propose that ruminating reflects hyperactivity and/or altered connectivity within the brain regions involved in self-referential and internally focused thinking, often coupled with ineffective modulation by cognitive control networks. Together, these patterns of dysconnectivity bias the brain toward persistent, inwardly directed processing of information.

Ruminating is associated with poor treatment outcomes across therapeutic modalities (67, 68). Due to the emerging insights about the clinical importance of ruminating, integrative understanding of this phenomenon is necessary (3). Phenomenology may thus provide an important component of an integrative understanding of ruminating (69). Qualitative insights have focused on lack of agency in ruminating, as well as a variety of themes that ruminations can be about (70–72). Social problems have been shown to be one of the most common triggers of ruminating as well as worrying about past mistakes (73). Distraction has been identified as the most common emotion regulation strategy people use to cope with ruminating. Rogiers et al. (74) reported that in the context of group-based therapeutic interventions, social support as well as the beneficial effects on their social functioning emerged as the dominant themes. Finally, in a recent study by Ciobotaru et al. (75), central themes were recurrent narratives about traumatic and painful past events, low self-worth, struggles to obtain mental wellbeing, isolation and mental stigma, as well as the all-encompassing nature of ruminative experience.

These qualitative insights, while valuable for our understanding of ruminative experiences and giving a voice to patients, have not followed contemporary developments in empirical phenomenology (76–79). Methodologically, contemporary qualitative phenomenological methods do not focus on the “what” of the experience (i.e., the content or what the experience is about) but on the “how” of the experience (i.e., in what mode of givenness a specific aspect of experience is present to consciousness). Qualitative phenomenological studies have not yet systematically investigated the fine-grained, moment-by-moment experiential dynamics (80) of ruminative episodes and the way in which ruminating reorganizes a person’s broader lifeworld (81). Building on recent developments in empirical phenomenology (76, 78, 79), the micro-phenomenological interview enables access to subtle experiential processes that can be linked to specific cognitive mechanisms. By experiential microdynamics, we refer to the subtle unfolding of rather than to global summaries or retrospective labels. Micro-phenomenological interviewing elicits detailed descriptions of a single episode (e.g., a specific bout of rumination) and then reconstructs its temporal structure: how attention shifts, images and memories arise, bodily sensations intensify, and evaluative thoughts loop or escalate. This allows us to describe experiences not just as a static “states,” but as a dynamic sequence of steps that aggregate into a characteristic pattern (82). Simultaneously, lifeworld analysis allows us to describe how these processes are embedded in and reshape patients’ baseline way of being-in-the-world, rather than treating ruminating merely as a maladaptive cognitive style.

This “empirical turn” in phenomenology has revealed several subtle phenomena that remain underexplored in phenomenological psychopathology. Huron (83), for example, speaks of *micro-emotions*, brief and difficult-to-catch affective experiences that can transform a sad piece of music into a pleasurable experience. Petitmengin et al. (84) showed that the experience of thinking is in and of itself rhythmic and has a strong embodied component. Various subtle mental acts have been shown to be able to radically alter one’s form of consciousness (85). So-called *attentional dispositions* refer to different styles of focusing, as reflected in lived experience, that are able to rearrange what is saliently present in one’s lifeworld (76, 86, 87). Finally, other than typical sensory illustrations of thinking (e.g., visual, auditory component), modes of thinking such as transmodal experiences (i.e., experiences with a common experiential core that can be translated into various other modalities) (84) and unsymbolized thinking (i.e., content in absence of modal illustration) (88) have been reported. It may be that there are experiential nuances to ruminating that have heretofore gone unobserved, and whose deeper understanding may help us alleviate our patients’ distress. Micro-phenomenology may be particularly suited for studying ruminating because its diachronic focus on moment-by-moment unfolding allows us to track how a single episode recursively loops, intensifies, or shifts in tone and content. While ruminating appears repetitive at a coarse-grained level, different episodes or cycles within them might be underpinned by distinct micro-dynamics. Similar uses of micro-phenomenology in other domains have shown that apparently unitary experiences, such as headaches (89) and insights during mathematical problem solving (90) involve highly differentiated temporal micro-dynamics that can be systematically described and compared.

Our research goal is therefore to provide a phenomenologically nuanced description of the lived experience of ruminating in patients with affective disorders and participants recruited from the normative population. We hope to a) provide clinicians with a better understanding of the patients’ lived experience of ruminating; and b) generate the breadth of possible experiences that ground ruminating that might generate testable hypotheses for future studies.

### 1.1 Reflexivity and organizing framework

Our primary motivating goal behind this study is our everyday interactions with others. By way of reflexivity statement, we wish to acknowledge that our primary place of work is a psychiatric clinic and many of us work as clinicians, as psychiatrists [LKK, JB, BŠ], a clinical psychologist [SR], technicians with transcranial magnetic stimulation (TMS) [AO, NK, JB], and teachers in higher education settings [UK, JB, BŠ]. We are daily met with people who are burdened and perplexed by their experience of ruminating, by how they are unable to stop doing it, and how it impacts their entire lives. Many of us can report vignettes from before this project began that motivated our interest in engaging with ruminating as an object of inquiry.

AO, NK, JB: In TMS treatment, patients often report that despite dysphoria generally abating, ruminations tend to persist. One patient, for example, reported that even though she initially felt better after treatment, she (being educated as a psychologist) could not stop thinking about how she scores highly on neuroticism, and how this is a stable trait that cannot simply be zapped away. Another patient reported that she felt better, which, in turn, changed her core-self narrative of “being a melancholy person.” She was unable to reconcile her mood and self-narrative. This ultimately prompted her to discontinue the treatment.

BŠ: I have often encountered ruminations in people who obsessively question everyday matters. I recall a younger patient who constantly wondered about and was preoccupied with the passage of time. When with other people, especially relatives and friends, he would think about how they would die, how they still had a certain number of years ahead of them, and then they would be gone. These thoughts about the passage of time - which is relentless, affects everyone, and leaves no room for comfort - would plunge him into sadness and tears. For him, the questions about time are ontological. He wonders about cosmic time, divine time. For him, they pertain to the very nature of the world.

UK: Two students reported on varying experiences of mind-wandering. The first one reported on how while she is ruminating, the contents of her thoughts feel “true.” When she is not ruminating, however, she reflects on them with a sense of disbelief. Her ruminations did not have logical rules or narrative structure and were manifestly untrue. On the other hand, another student reported that she does not ruminate. In fact, she does not mind-wander! She experiences a tendency towards mental silence. This causes her problems (e.g., at one point, she got so absorbed in mental quietude that she almost crashed her bike). Thus, she tries to ruminate on purpose, but this requires a lot of effort.

All the authors: Recently, our group conducted two cases studies of individuals who exhibit extreme forms of ruminations: Hernan (91) and Benjamin (92). They both alot a significant amount of time to their self-reflection, trying to construct clear-cut, logical rules about how they work as human beings. For Hernan, this takes the form of elaborate ruminative rituals. Benjamin, on the other hand, wrote five book-long texts of self-reflection. We conceptualized this organization of their lifeworlds as “being-in-interpretation.”

The organizing framework of this study is thus based on our interactions with these individuals. We seek to provide a phenomenological account of ruminative experience, the lifeworld it inhabits, and the worldview it motivates.

The term *phenomenology* is used with three broad meanings: a) a coarse-grained description of symptoms; b) a qualitative practice of collecting data on people’s experiences; and c) a school of philosophy that attempts to disclose the fundamental structures of consciousness (93). While useful for rapid clinical communication, most phenomenological accounts of ruminating (for a review, see (94)) are limited to loose descriptions of symptoms, precluding a deeper understanding of this phenomenon. Our phenomenology is qualitatively oriented. Here, we are following the recent empirical turn in phenomenology that is attempting to ground theorizing in participants’ subjective reports rather than abstract philosophizing. Empirical, qualitatively focused phenomenology commonly adopts a position of *radical beginnings* (95) or *theoretical naivety* (76, 96, 97) wherein it attempts to observe preexisting concepts and theories from a fresh perspective thereby reexamining their validity (98, 99) as well as including the voices of people who are experiencing a given phenomena (see so-called *epistemic justice* (100)). We have previously validated the most standard clinical questionnaire for estimating ruminating, the Ruminative Response Scale, into Slovene (25), where its psychometric properties have been shown to be poor (we observed the same problems as (24)). Thus, we wanted to steer clear of pre-existing frameworks and focus on individuals who, on their own terms, experience ruminating.

Nevertheless, we adopted two philosophical ideas to constrain our analysis: *lifeworld* and *worldview.* Such integration of qualitative and philosophical phenomenology is typical in the field of phenomenological psychopathology (101, 102). In phenomenology, lifeworld [Ger. *Lebenswelt*] is the world as it is uncovered in our experience prior to any scientific theorizing. It is the dynamic background against which our lives take place (103). Lifeworlds are shaped by our broader existential conditions, both material (e.g., our socio-economic background) and imaginal (e.g., our typical psychological functioning). They appear as integrated experiential wholes [Ger. *Gestalten*] that allow for a pragmatic orientation in the world (104). Lifeworlds are comprised of our prereflective attunement with the environment (An. Gr. *koiné aisthesis*) and the culturally received knowledge that allows for taken-for-granted engagement with others (Lat. *sensus communis*) (105). Such integrated descriptions are meaningful because they allow us to make sense of patients’ psychopathology as it appears in the broader context of their lives, rather than analytically derived scientific constructs (101, 106). By looking at a person’s lifeworld, we are no longer talking about depersonalized clusters of symptoms. Rather, we are geared towards people as wholes, how disorders affect their whole existences (as opposed to simply claiming that they lead to heightened dysphoria) (107). Such a perspective is essential as it allows both for better therapeutic contact with the patients by fostering empathy (107, 108) and the development of existentially oriented psychotherapeutic interventions (109).

Jaspers’ (110) notion of worldview [Ger. *Weltanschauung*] refers to a person’s prereflective belief about what the world is and how it works. It is always derived from a person’s embodied, lived experience (111). Worldviews serve as housings [Ger. *Gehäusen*] that allow us to make sense of stressful and traumatic events and shield us from psychopathological responses. They foster *existential resilience* (112). Worldviews can fracture in limit situations (e.g., bereavement, major life transitions) (110, 113). For Jaspers, it is essential that in such circumstances we do not fall back on old, received, or simplistic worldviews, but construct them anew based on our experience. Worldviews are composed of two poles: the world pictures [Ger. *Weltbilden*] and our attitudes towards them. They do not amount to ideologies (e.g., liberalism, authoritarianism), but pertain to our ontological commitments. One might experience the world as deeply atomistic, prompting feelings of loneliness and isolation (as was the case for Wittgenstein during his depressive episodes (114)). Or one might feel the world to be deeply connected, with all of us being a part of a great chain of being, prompting feelings of unity and cosmic camaraderie (as is the case in mystical experience (115)). Given Jaspers’ dynamic conception of worldviews, it would be safe to posit that in ruminating, people attempt to construct a coherent worldview, but fail to do so.

## 2 Methods

### 2.1 Participants

We included the data from a total of 51 participants (forty women). Participants were on average 26.6 years old (*SD* = 7.1) and had completed 18.6 years of education (*SD* = 4.2). Thirty participants had formal psychiatric diagnoses. Since ruminating can occur both as a core and a peripheral symptom, and in both clinical and subclinical populations (35), twenty-one participants were recruited from the normative population in order for us to construct a more comprehensive account of this phenomenon. The sample size was determined using formal conceptual depth criteria (see more on this below). The clinical sample was recruited from out-patient program at the University Psychiatric Clinic Ljubljana based on whether they experience ruminating despite ongoing psychiatric treatment. We excluded patients with a history of psychosis or comorbid addiction disorders. The normative sample was recruited via a mailing list at the Center for Cognitive Science at the Faculty of Education at the University of Ljubljana inquiring whether people experience frequent ruminations that are a source of distress for them. Demographic data with specific diagnostic categories are presented in **Table 1**. All participants signed an informed consent to participate in the study. The study was approved by the Committee for Medical Ethics of Republic of Slovenia.

**Table 1:** Demographic characteristics. Diagnoses are specified according to the International Classification of Diseases, Tenth version (ICD-10).

Variable	Normative sample (N = 21)	Patient sample (N = 30)
Gender		
Men [*f*]	0	11
Women [*f*]	21	19
Age [mean (*SD*)]	24.6 (7.3)	28 (6.6)
Years of completed education [mean (*SD*)]	17.0 (1.9)	19.8 (4.9)
Primary psychiatric diagnosis [*f*]		
F33 0 (Major depressive disorder, recurrent, mild)	0	5
F33 1 (Major depressive disorder, recurrent, moderate)	0	10
F33 2 (Major depressive disorder, recurrent severe without psychotic features)	0	3
F41 1 (Generalized anxiety disorder)	2*	5
F41 2 (Mixed anxious-depressive disorder)	1*	7
Comorbid psychiatric diagnosis [*f*]		
F90 0 (Attention-deficit hyperactivity disorder, predominantly inattentive type)	0	2
F60 3 (Borderline personality disorder)	0	4
F50 2 (Bulimia nervosa)	0	2
Therapeutic modality		
Psychotherapy	3	18
Pharmacotherapy	0	19

*Stable symptomatic remission.

### 2.2 Data collection and analysis

Since this study is a part of a broader research project on the phenomenology of emotion dysregulation (116–118) our starting point was archival qualitative material. Additional twenty-two participants were recruited to provide confirmatory data and further insights.

Data were collected with a phenomenological interview. The interview itself drew on two techniques: the micro-phenomenological interview (119) and lifeworld analysis (107, 108). Micro-phenomenological interview is a qualitative method that aims at collecting fine grained description of experience on a moment-by-moment basis. It focuses on detailed descriptions of a moment-by-moment of experiential dynamics (80). First, the interview opens with an evocation phase where a well-defined experiential episode is selected and described in as much sensory detail as possible. Second, a diachronic description of experience (i.e., how it evolves in time) is collected. Third, a synchronic description of experience (i.e., a detailed description of specific moments) is collected (120). Both at the level of conducting the interview and analyzing the qualitative material, so-called satellite dimensions of experience are removed (e.g., descriptions of non-experiential scientific or folk psychological theories). Rather, descriptions of sensory experiences, bodily feelings, as well as mental gestures (119) are favored. Micro-phenomenological interviews yield detailed enough data to allow for theorizing about the associated cognitive processes (80) as well as operationalization in narrowly defined research designs (e.g., using neuroscientific methods) (121). However, as has recently been pointed out, psychopathology does not bear only on specific moments of experience, but draws on people’s broader existential situation as well (81, 122). Thus, we complemented the micro-phenomenological interview with lifeworld analysis.

Lifeworld analysis is a method that has been developed for phenomenological psychopathology. It aims at describing a patient’s baseline way of being in the world [Ger. *In-der-Welt-sein*] (123). It reduces experiential reports to the descriptions of body, space, time, emotions, the experience of others and a person’s values. Unlike micro-phenomenology, lifeworld analysis assumes an engaged interviewer who empathizes with the patients by asking specific questions to attune themselves to their experience. This proved essential as our participants were initially reluctant to talk about how they conceptualize themselves in their ruminating. Rather than focusing on a moment-by-moment experience, lifeworld analysis yields a person’s typical experiential Gestalt. It incorporates phenomenological accounts of experience as well as the patients’ ways of interpreting their experiences (i.e., it adopts a hermeneutic approach) and how stable aspects of their lifeworld evolved through various life stages (i.e., it has a psychodynamic component). Thus, it incorporates descriptions of experiences that are inadmissible under micro-phenomenology (e.g., probably unveridical phenomena such as childhood memories and metaphorical descriptions), which are nonetheless meaningful when trying to understand psychopathology.

**Figure 1:**
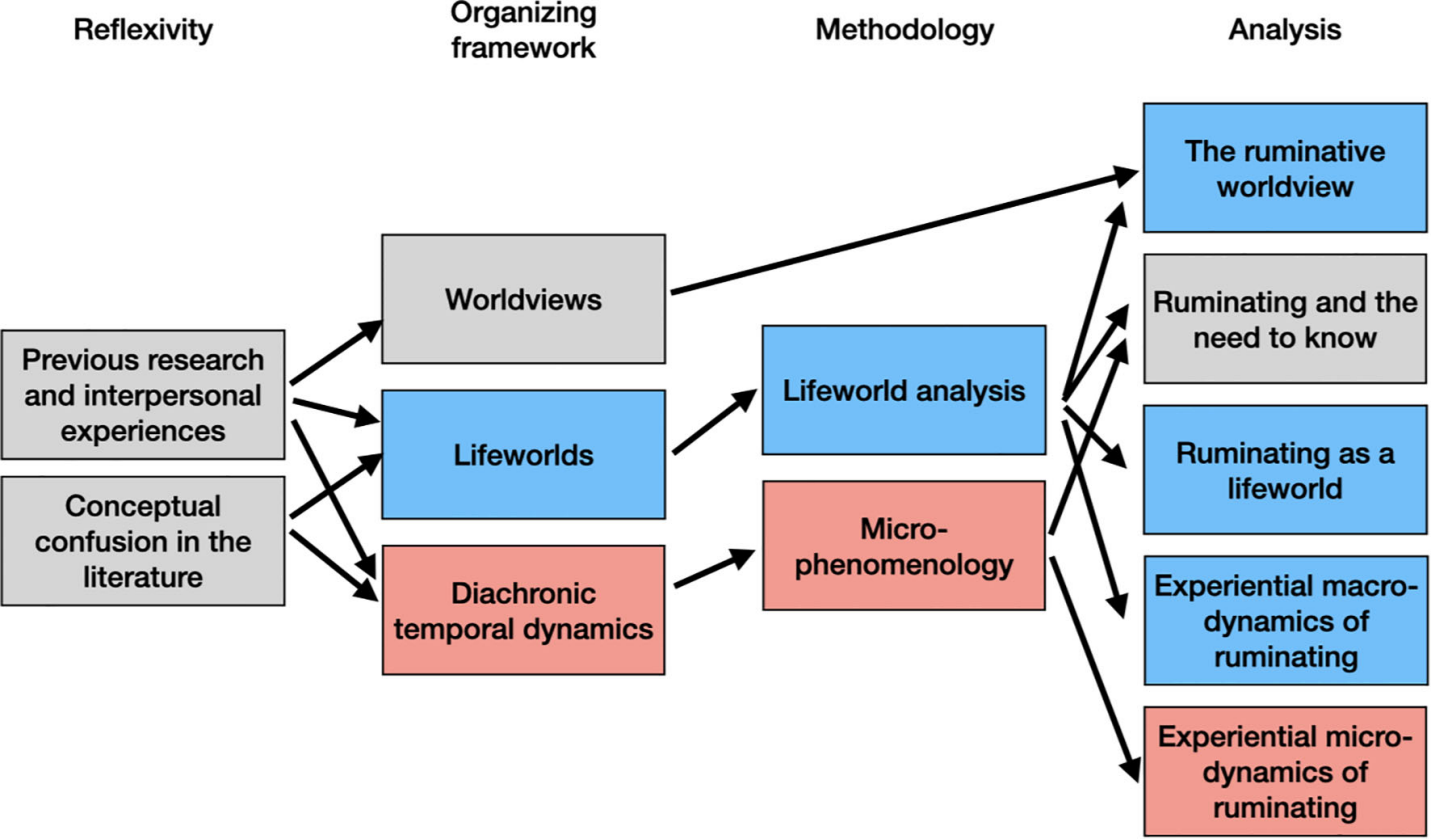
Method construction. The diagram shows how our reflexive positionality informed our organizing framework, which in turn informed method selection and analytic induction of categories. Blue/red hues denote direct basis, whereas gray includes interpretative leaps.

Practically, the interviews followed a structured, but processual pattern (i.e., rather than imposing a strict sequence of questions, we followed our participants in how they disclosed specific details). We asked the participants to recall a particularly intense episode of ruminating. First, we asked them to situate the episode in their broader autobiography (what was their existential, material situation, what were their main preoccupations and sources of distress). Then, we moved on to “how it felt to be them” while ruminating. We focused on the domains of lifeworld analysis mentioned above, while paying particular attention to collecting contrasting descriptions from within and without a ruminative episode. Finally, we focused on the specificities of ruminating. We employed micro-phenomenology to collect descriptions of ruminating as it appeared in the moment, which, in turn, could then be analytically contextualized in their broader lifeworld at the time. It is to be noted that within the substructure of the micro-phenomenological interview, participants occasionally referred back to the description of their broader lifeworld. Thus, in the analysis (which will be described in more detail below), some overarching themes could be relatively clearly related to the specific methodological framework, whereas one theme (described in subchapter “Ruminating and the need to know”) was derived from both. **Figure 1** summarizes the flow of how our previous scholarly and interpersonal experiences are related to our organizing framework, how this informed our methods of data collection, and finally, how they lead to different angles of impact into understanding ruminating.

The interviews were conducted by two researchers formally trained in qualitative research broadly and clinical interviewing specifically [AO & SR]. The researchers have collaborated on the broader project on the lived experience of emotion dysregulation since 2022 and have harmonized the interviewing style such that a comparative analysis can be made on the pooled reports. The data acquisition and analysis processes ran in parallel (124). The data were analyzed jointly by multiple researchers [AO, SR & NK] using coding; that is, the process of assigning more general descriptive tags to sections of raw text based on their descriptive similarities (124). Three forms of coding were used: inductive, inductive-deductive, and deductive coding (125). Initially, we used inductive coding where categories were derived in a theory-naïve way directly from our participants’ reports. Second, inductive-deductive coding was used. As categories emerged, we fitted them onto data from newly recruited participants. However, we kept an open mind to where categories that were established previously could be reconceptualized or abandoned in favor of better fitting categories. As the final taxonomy emerged, we used deductive coding to go through all the interviews again and fit it onto the data.

The validity of the data analysis was first established through intercoder verification. The final taxonomy was refined through iterative discussions among the authors, during which differing interpretations were examined and consensus on theme definitions was reached. Instances of codes that did not fit emerging categories (i.e., negative or deviant cases) were explicitly sought out and used to challenge and, where necessary, revise or nuance definitions (126, 127).

**Figure 2:**
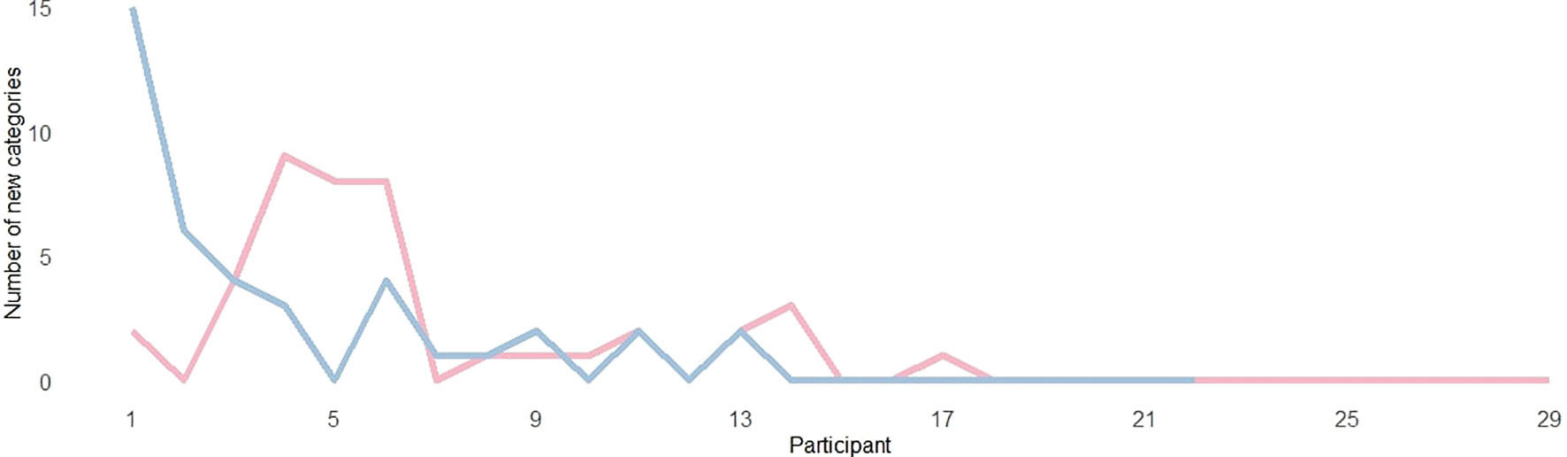
Estimation of conceptual depth. Archival data (red) and newly collected data (blue) are estimated separately.

The second measure of validity of the data was an explicit estimation of conceptual depth (also known as saturation): the point at which we collected a sufficient amount of data to allow for theory construction (128, 129). It was estimated with a saturation grid, a tabulation wherein the number of newly observed categories is assigned to each newly recruited participant. In the archival dataset, no new codes were observed after the 17th participant. In the newly collected data, no new codes were observed after the 13th participant. **Figure 2** summarizes the saturation grid. The observed categories are described and supplemented with quotes in a codebook (available at: https://osf.io/3xnmu/).

## 3 Results

**Figure 3:**
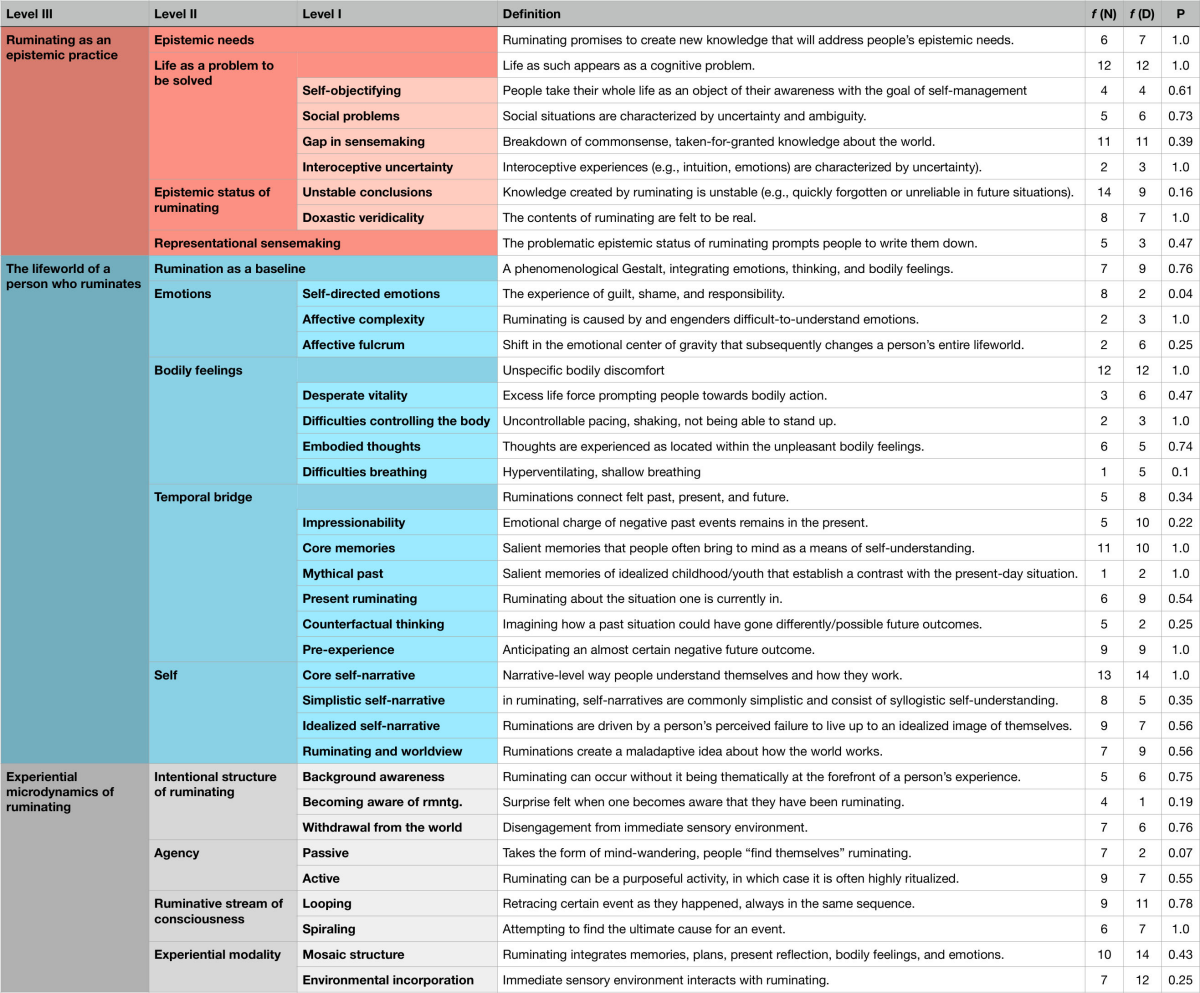
Taxonomy of experiential categories. *f*(N) denotes in how many participants from the normative sample a given category was observed, whereas *f*(D) denotes in how many participants from the clinical sample a given category was observed. Differences were compared using Fisher’s exact test. Frequencies denote how many participants reported on a given category across all the investigated experiential episodes. The frequencies should not be interpreted as reflecting epidemiological data but rather the conceptual depth of individual categories.

107 interviews (on average, 2.1 per participant, *min* = 1, *max* = 11) on 79 experiential episodes were conducted. The interviews were on average 52.6 minutes long (*SD* = 12.3). The qualitative analysis yielded 39 experiential categories that were organized into six superordinate themes, each including three levels of coding. These are summarized in **Figure 3**. Due to the recent methodological discussion in the field of qualitative phenomenology wherein different methods (co)determine the range of possible findings (76, 130), the highest level of coding reflected the two styles of interviewing we employed.

### 3.1 Ruminating and the need to know

Participants consistently described ruminations as a process of creating knowledge aimed at reducing self-related uncertainty. One patient felt them to be a “development of events in which I actually, um, have access to learn more.” For another one, they represent “moments of epiphany.” A participant from the normative sample similarly describes them as follows: “The personal throughline was this search for reasons. [ … ] it’s as if it’s revealed to me by God.” Another patient describes ruminating as going “into scientist mode,” while another one employed the metaphor of “waving away the fog.” As such, ruminating can be understood as addressing a person’s need to know.

A patient spent a lot of time imagining her boyfriend cheating on her. While her ruminations were painful, she also derived satisfaction from them:

Definitely the need to get to the bottom of things. That feeling of relief, when, while studying, you sort of figure something out and things click into place, and everything integrates correctly. [ … ] Just proving that I know what’s going on.

Ruminating was commonly triggered by the collapse of a common-sense understanding of the world. People’s lives stop being something that is to be lived spontaneously. They become an intellectual dilemma, a problem that is to be solved. As such, ruminating is characterized by a problematic relationship with knowledge. Conclusions of ruminating were often poorly supported by evidence (e.g., interpreting a coworker’s sigh as reflecting their poor job performance). For example, a medical doctor suddenly started worrying about using an unsterilized tool during surgery:

I always felt like I had everything under control. [ … ] Then, all of a sudden, we were getting ready for a procedure, and I opened up everything sterile and put it all out on the table for the surgeon. [ … ] After I’d set everything up, it hit me that maybe I hadn’t put out a sterile tray. But there was a label on it saying it was sterilized. [ … ] And then the procedure happened, and it was fine. [ … ] I got home and I spent the whole evening, and even the next few days, thinking about what might happen to that person now. [ … ]*What should I do? Should I say something, should I not say anything? Would it even change anything if I did?*

The content of ruminating is epistemically problematic. Commonly, our participants ruminated about entirely fictional situations. When ruminating about a mistake, the perceived punishment can be overblown (e.g., a patient was not only afraid that his mistake would cost him his job but that he would become homeless because of it). Similarly, ruminations can overblow perceived burden of responsibility. Yet, these hypertrophies of meaning feel real. However, the knowledge it produces is unstable (e.g., a person might forget what they had learned, or the resulting knowledge might prove counterproductive in future circumstances). As one participant remarked: “I’m going to really think about it and maybe I’ll discover something new. I absolutely never discover anything.”

Ruminating is thus associated with an ongoing (re)construction of a person’s self-understanding as flawed. Ruminative self-understanding is simplistic, often reflected in statements such as “I’m just way too much,” “I am stupid,” ‘I’m a bit slow,” ‘I’m weird,” and “I’m arrogant.” These ideas about the self-integrate with the sense that the content of ruminating is real. “*Nobody likes you*,” a patient described the conclusion of her ruminations, before adding *sotto voce*, “This is the truest thing.”

In ruminating, people narrate themselves not as complex beings capable of processual reasoning and adaptive behavior, but construe themselves as poorly written characters, incapable of navigating their lives, and who are deterministically bound to repeat their own mistakes. As one normative participant described it, in light of this story, “the radical hypothesis” that she did the worst possible thing in an interactio makes sense.

### 3.2 Ruminating as a lifeworld

Ruminating amounts to a pathological way of being-in-the-world, integrating altered experience of embodiment, temporality, and emotions. As one patient noted: “The thing was that it was ever-present. [ … ] Everything became about this feeling”. Another patient reported: “I literally feel like I am my thoughts, rather than just being a person”. Similarly, a patient with mixed anxious-depressive disorder reports: “Rarely do I feel that I am not overthinking.” Another patient with anxiety sullenly described her ruminations as follows: “Three-quarters of my energy for functioning in the world is drained by these, um, often overly burdensome thoughts.

Many patients wake up into ruminations: “I ruminate all the time [laughs], especially in the morning I think that the world should just collapse.” While patients often remarked that ruminating constitutes a universal aspect of experience, recognizing that there are other ways of being can be beneficial to their mental health: “I figured out on my own that this isn’t normal. Not everyone functions like this. It helps me to see that there are people who do the same things and don’t even worry about it.

Ruminating is both triggered by and generates negative self-related emotions. These primarily consist of guilt, shame, and responsibility (experienced as an expectation of punishment). Situations that cause ruminating are characterized by mixed or ambiguous emotions. People often do not know how to feel about a situation, nor can they rely on their intuition to make sense of it. Ruminating is deployed in an attempt to make sense of this complexity: “I need to do this for a while now and pay attention and energy to diagnosing what I feel, because I am not entirely sure I know what I feel.”

Ruminating bridges past, present, and future, linking emotional events across time and fostering counterfactual thinking and anticipation of future difficulties. Thus, a neuroscience student who had just learned that his father had been diagnosed with Parkinson’s disease and having been put in the role of an “expert” during the first family conversations about the situation:

I was making some predictions, some guesses about how I thought all this would turn out. [ … ] I even thought about the conversation with my mom, which was really kind of intense and full of content from both sides, especially hers. I kind of knew … even though I thought something similar would follow [ … ] I was telling myself, *okay, I’ll say this, then I won’t say anything, then I’ll say that*, and then again nothing you know, in those potential predictions.

Ruminating is commonly triggered by the emotional charge of past events remaining salient into the present. Consider the following report by a participant who was dancing at the club and noticing that a man and a woman were constantly looking at her. The stranger had made a gesture that the participant related to both drug users as well as a memory of her mother who had been diagnosed with bipolar disorder:

It feels like I had this strong emotional response and I was trying to make sense out of it, and um my mind was going through what the reason might be and then I had this sense that they’re plotting something terrible and I think maybe at some point I even had like this explicit thought about maybe they want to kidnap me.

Ruminating is commonly associated with a decreased motivation for productive problem-solving. We observed that it was the anticipation of the future that deprived our participants of agency:

That aspect that there’s a bit of [pause] this kind of rotten situation, again! Over and over! [ … ] That “no way out” feeling. [ … ] I really strongly feel this sense that there’s no solution.

Finally, ruminating is reflected in the body. Breathing becomes difficult. Intense ruminating is associated with feelings of tension and tightness, most typically in the chest, prompting participants to report that ruminations have a location in the body. Two patients employed the same metaphor of “thoughts burning in their chest”. Furthermore, participants report that their bodies become difficult to control, an experience that is commonly grounded in shaky knees, ticks and twitches, and muscular tension. Ruminating is sometimes associated with desperate vitality, an excess of lifeforce, where outbursts of energy force patients to enact sometimes inappropriate impulses (131).

### 3.3 The ruminative worldview

Above, we noted that the knowledge that is produced by ruminating is unstable. This fleeting nature of the ruminative results engenders the need in people to write them down:

The closest I can get to drawing conclusions is when I write something down. [ … ] Because then it’s black on white, and I can come back to it. Otherwise, I can’t say that I ever really conclude anything in my thoughts.

Similarly, a patient with anxiety:

I left some proof of my mental activity there. That was an important response. And I started writing the thing. [ … ] Something has to remain. Either on paper or on the computer.

One participant has moved beyond journaling to using a large-language models:

I’ve actually developed a new routine where I talk to ChatGPT every day and feel like I’m addicted to it. [ … ] I don’t feel like a burden to people, since if I keep analyzing the same situation over and over, I feel like they’re getting tired of me. But ChatGPT is always here for me [laughs].

While journaling has typically been conceptualized as an adaptive form of ruminating (132) our findings suggest that they generate an external reminder of one’s perceived shortcomings. One patient, a PhD student, would sit down to write her thesis. She kept three mediums to keep a record of her mental activity: a physical diary, a journal on her computer, and an Excel spreadsheet. As unpleasant thoughts started to emerge, she would switch tabs and start writing down her ruminations:

Something takes my attention, and I need to explain something. Then I open the document and switch to the diary and then I write down what worries me, why I blame myself, or … um … an explanation of why something happened. [ … ] There is this feeling that it literally went out of my brain for a while. [ … ] I externalize part of these ruminations for a short time, and I can focus again.

While this method may initially seem productive, her journal entries contain entries such as “I won’t eat today.” Thus, writing down ruminating resolves the uncertainty of overthinking by creating evidence of what people had thought about. But it also creates an externalized scaffolding that can deepen psychopathology. This cyclical structure is the fundamental aspect of the ruminative experience. People become thematically aware of their whole existence, rendering it an object that can be manipulated and optimized:

I go into this mode of manipulating, optimizing, rationalizing myself. I don’t see anything. I am just describing in inner speech things that are going on. There is this narrative in my mind. I don’t really feel well-placed in the world. [ … ] My life becomes a story in my mind.

Through the construction and repetition of these narratives, ruminating creates a specific worldview, integrating a person’s lived experience, psychological, and folk scientific concepts:

There was this moment of finding something out about my life. I am saying that this was “spiritual” or “astrological” because most of the meaning that I find outside of myself comes from this area of reading about it. [ … ] It was pragmatic. It has to do with my life, how I am perceiving my level of satisfaction oscillating. I know it has to do with hormones and neural transmitters. [ … ] It was basically me trying to create these little explanatory stories about the world. [ … ] The context is usually constructed from these astrological or spiritual contents. [ … ] I reach this aha moment, where I feel like I figured out a truth, a truth about life, something that liberates me from this constant anxious feeling.

Ruminating is a self-reinforcing cycle, in which one’s emotions are interpreted as truth, and that truth keeps proving that the self is flawed. In this circular process, emotional states and negative self-appraisals continuously reinforce one another.

### 3.4 Experiential micro-dynamics of ruminating

#### 3.4.1 Polymorphic structure of ruminating

**Figure 4:**
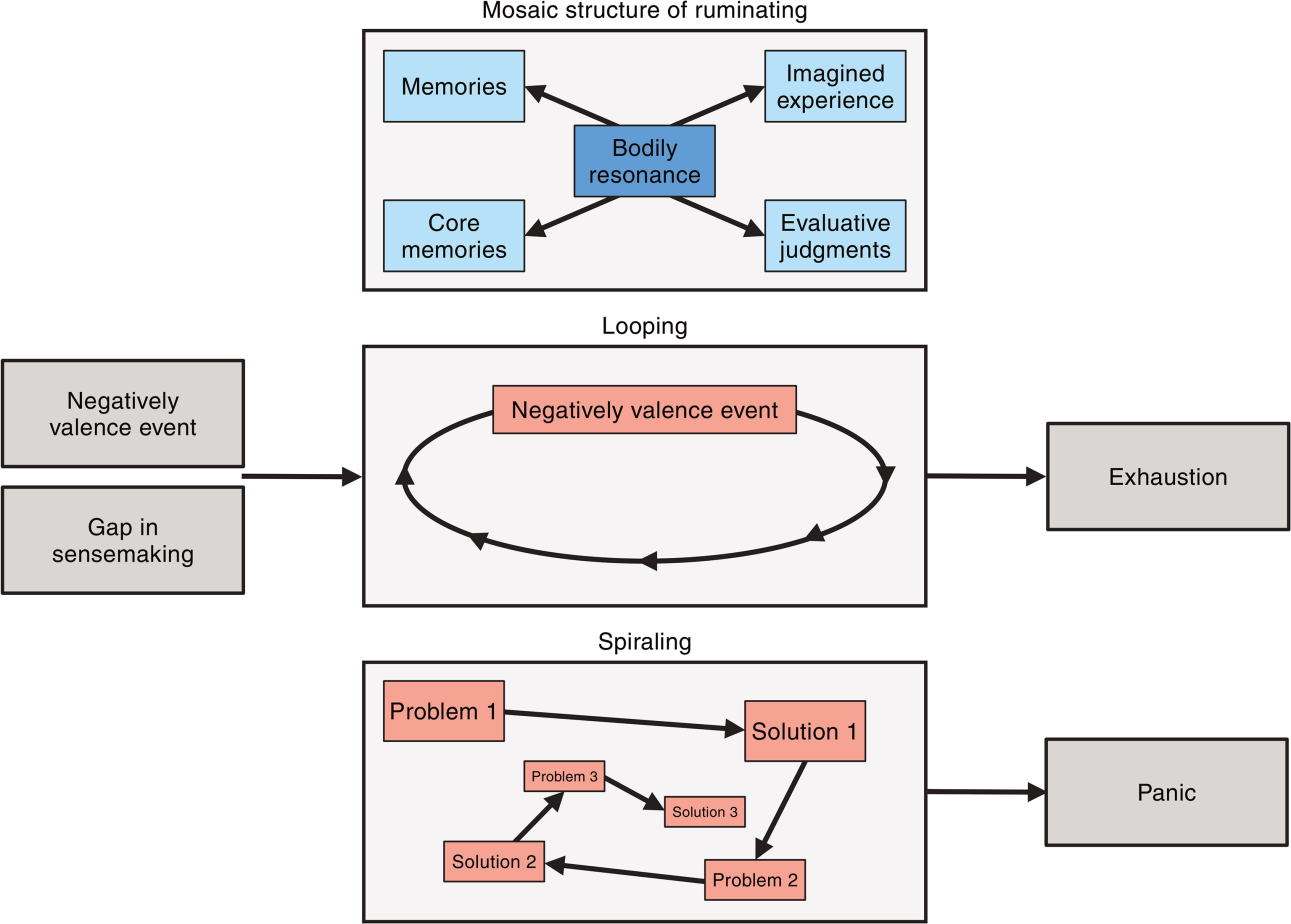
A schematic representation of the diachronic structure of ruminative experience. Mosaic structure of ruminating is depicted in blue. Looping and spiraling are depicted in red.

**Figure 5:**
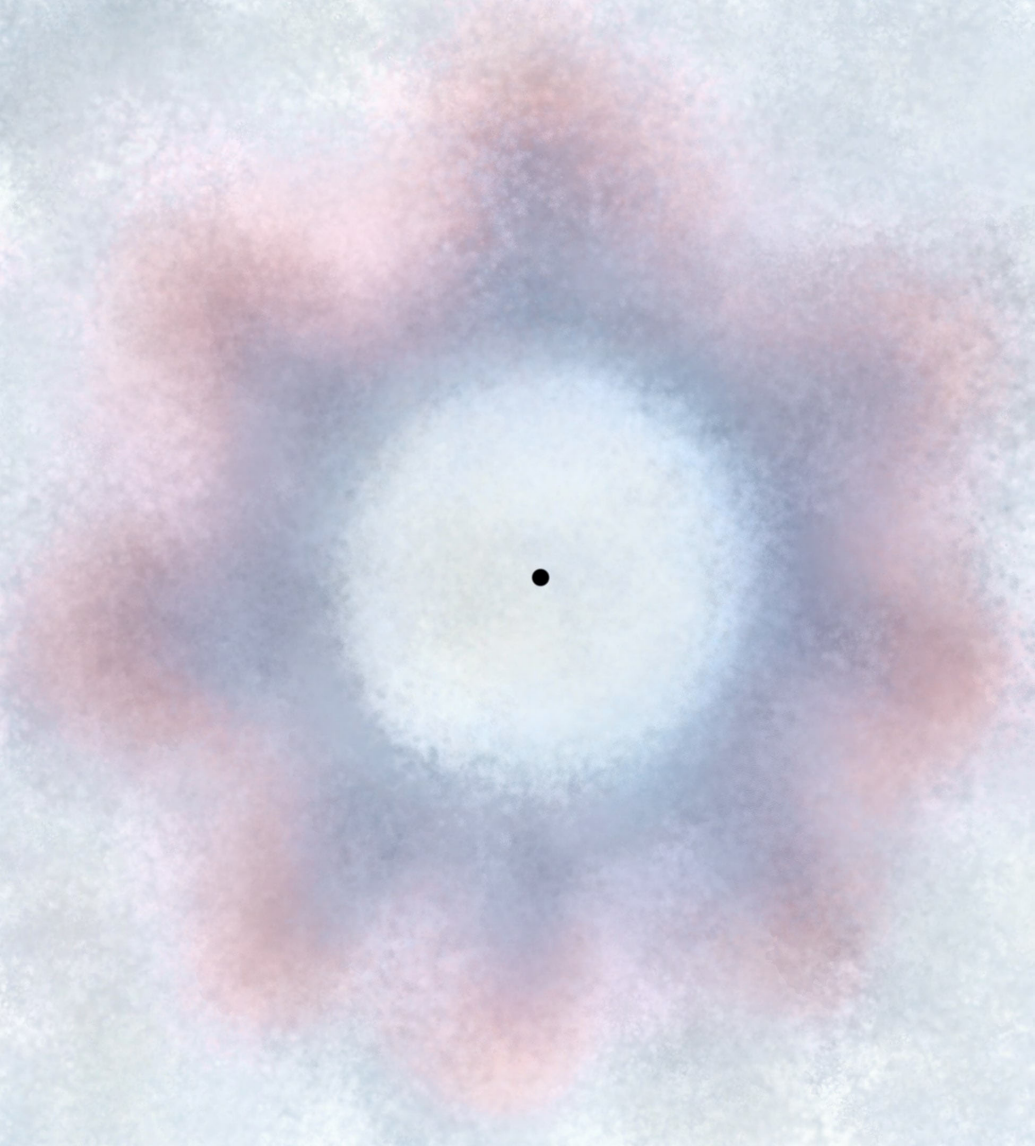
A reproduction of a drawing made by a patient. Her comment was as follows: “I somehow imagined stimulus and response and the time, the space in between, which is for you to react. Basically, the stimulus was made with a small dot and around it was an emptiness. [ … ]. I also wanted to show that I made these contrasts with colors. Blue is some benign thing when you start dealing with the problem. With red, I wanted to emphasize the chaos a little, which remains and can drag on. That’s how I wanted to clearly show it.

One of the two methodological frameworks that we employed in this study was the micro-phenomenological interview, which aims at collecting reports on micro-dynamics of lived experience at a moment-by-moment basis (80). The resulting experiential microdynamics is depicted in **Figure 4**. Two aspects of ruminative phenomenology were particularly salient. First, ruminating is a polymorphic experience. It has a mosaic-like structure with memories, imagined experience, evaluative judgments, etc. blending together into a single, unified stream of consciousness. Thus, a patient with depression: “It all happens at once, but … I feel it as different parts. I can feel the cognitive part and the tension separately.” Similarly, a participant from the normative sample: “It’s not just one feeling that I feel alone in that previous situation, but it’s like a conglomerate. [ … ] It’s its own feeling, which contains all of that.” One participant described ruminating as being structured like a “network”. A patient reproduced a drawing she had once made in her diary with watercolors to represent the mosaic structure of her ruminations (see **Figure 5**).

Participants commonly reported on the presence of core memories; that is, memories, typically from childhood they tend to recall to understand their broader existential situation. People ruminated about situations that had a similar emotional charge as discord in their primary family. References to “parents”, “fathers”, and “mothers” were commonplace, as were difficult emotional situations from past romantic relationships. People recalled traumatizing experiences with professors from higher education and being ostracized in elementary or high school.

During conversations with a severely depressed patient, he would mention the promise of “ultimate humiliation” when he would be ruminating in social settings. It was not until the later interviews that he felt comfortable enough to share an event in elementary school when he asked the teacher whether he could go to the toilet. Being rebuffed by the teacher, he ultimately peed himself in front of his classmates.

Core memories need not be memories of traumatic events. People can reminisce about a mythical past when they were happy or well-adjusted:

I guess just the general [pause] way of how we spoke to each other. [ … ] We could all talk to each other, and there was like no conflict between classmates. [ … ] It was just easygoing.

It is the bodily resonance of these disparate components that provides the throughline for ruminating, constructing a uniform experience:

Everything is collapsing inward. [ … ] It is all emotional! But actually, it is physical. [ … ] The physical feeling contains this content. It is emotional, mental, physical. Everything is blended together into this one thing.

Ruminative mode of experience is further characterized by environmental incorporation. Certain contexts appear more conducive to ruminating (e.g., going for a walk, driving a car). As one participant remarked: “At home, it seems like it gets the most intense, probably because things calm down a bit and I start thinking more”. Specific music can provide the context for ruminating: “Angry-feeling music. For example, Rammstein, or, if you go into some nostalgia, Linkin Park when they were screaming a lot”. A patient spent weeks reliving a difficult social situation with her partner and, at the same time, planning a resolution of the relationship by going for a walk in the same forest path she had taken with him. “It’s somewhat related to the specific geography,” she remarked, “There you are a bit limited..”

#### 3.4.2 Looping and spiraling: two ruminative streams of consciousness

The second experiential micro-dynamics that we observed refers to the difference between two ruminative streams of consciousness: *looping* and *spiraling.* They differ in terms of how people move from topic to topic while ruminating, and what is the felt “pseudophilosophical” motivation behind them.

Participants reported that in looping, they *replay* specific a situation in a relatively fixed way, almost reliving it as it happened:

I am located in that reliving. It is almost as if the only way I can relive this situation is exactly in the way that it. […] It was programmed. I don’t need to enact it. I just automatically draw on that situation. [ … ] I am performing what happened then.

They return to the same scene, details, and interpretations. One participant described such memories as “scripted.” Another patient used the metaphor of waves moving against a shoreline. She would have a thought, reach a conclusion, but a similar wave will come, repeating the cycle into infinity.

Looping exhibits a hermeneutic attitude. People recognize that experiences cany be viewed, analyzed, and interpreted from different vantage points. Thus, a patient: “I interpreted how I felt on that day. [ … ] Thirty different ways of interpreting it. One interpretation per day.” In looping, the contents of what will be thought about are typically known in advance: “I feel like many times I already know in advance what the outcome will be. [ … ] It’s usually a pretty accurate assessment of what will happen in the end. But I still go through the process.” Sometimes, patients engage in looping as a form of mental self-harm, as torture by reflection: “I was actually torturing myself. [ … ] It was almost as if I wanted to be punished a little bit. *You basically didn’t do anything yet. Let’s go at it again.”*

On the other hand, participants reported on spiraling. People think about an existential problem, find a solution to it, only to realize that the “actual” nature of the problem is deeper. They are like scientists attempting to uncover an increasingly fundamental structure to their problems, only for new underlying contents to emerge.

In spiraling, the philosophical motive is ontological. People try to find the ultimate causal mechanism of their distress. They are faced, again and again, by the realization put forth by Sartre (133) that at the heart of human life, there is no ground truth, only nothingness:

I felt like: “Okay, I am on the road now. I am observing it and approaching his pain.” And it ended suddenly. Bam! With each new realization that I reached, the subsequent association kind of [pause] clashed with it. With each click, there was something new. A new package of weight loaded onto me.

Whereas in looping, our participants report that there is an endpoint where they have exhausted the amount of information afforded by a specific situation, in spiraling, there is no such point. If spiraling is not interrupted, it can lead to a panic attack: “I get stuck in that loop and it just escalates. [ … ] It’s a state where I’m completely, completely panicked and I can’t see a way out of the cycle.

A patient reported that at the end of spiraling (especially if it happens in social situations), there comes a state of complete passivity:

There are just so many thoughts in my mind. Every thought relates to every other thought. Every thought opens more thoughts. It feels as if I cannot find some stable anchor that would allow me to do anything. Mentally or physically. It’s difficult to explain but because I cannot think through all of them, or even one of them, I cannot do anything. It feels like I am being strangled by this thick mental snake.

He concludes:

At the bottom of it all, every potential angle of impact just feels so wrong that I cannot even think about anything anymore. There is just this hopeless passivity. This doubt about literally everything. [Chuckles dryly] I think that’s what Nietzsche meant when he talked about the void staring back.

### 3.5 Experiential macro-dynamics of ruminating

Much like micro-phenomenology uncovers the micro-dynamics of lived experience, lifeworld analysis, by focusing on a person’s baseline way of being-in-the-world is able to uncover the macro-dynamics of lived experience. Several participants reported on how they felt ruminating triggered a depressive episode. Here, we will present a *genetic analysis^1^* of this experience. Ruminating is felt to be an affective fulcrum. By this term, we refer to the emotional center of gravity that organizes one’s inner experience. Rather than being a passive background process, it is experienced as actively structuring the sense of time, space, self, and existential orientation. This dynamic was best characterized by a participant who had long struggled with depression but has been in stable symptomatic remission for two years at the time of the interviews. He reports moving abroad for his PhD and facing bureaucratic difficulties. Gradually, he started to ruminate on these difficulties, leading to self-narrative about being incompetent:

It really reminds me of the early period in undergrad, where there was just horror, where we had this planned ego-crushing moment, when I started asking myself, do I even deserve to live, am I even good enough to live. [ … ] When I don’t have things under control, my desire to even want something starts to wither. [ … ] In those moments of this bureaucratic war and loss of control, I’m deeply thinking about my years at university, or maybe unfulfilled first loves. They are somehow connected to that. [ … ] But that moment when you close yourself into that inner world. [ … ] I think, yeah, it causes me even more confusion.

In ruminating, his uncertain life obtained a coherent narrative structure:

At least some kind of narrative starts to form and there’s some thread, some continuous inner speech. *Okay, wait, calm down, you’re totally freaked out, you’ve really started to lose it, blah blah blah.* Um, so the form of this inner speech becomes much clearer, and at the same time it also becomes more coherent. There’s more of a thread, a more understandable narrative.

Finally, he felt that, in a cyclical manner, ruminations started to engender negative emotions:

It seems to me that I automatically start to experience the thoughts … or rather transform them in some emotional way. [ … ] I don’t need to interpret the content and then some emotional aspect comes out, but the content is automatically connected with the fact that some negative emotional experience comes along with it.

In this way, ruminating is experienced as shifting a person’s entire phenomenological Gestalt from euthymic to depressive.

## 4 Discussion

The goal of the present study was to put forward a descriptive account of the lived experience of ruminating. We collected qualitative material from patients with affective disorders and participants sampled from the normative population. One of the most salient aspects of the qualitative material was that ruminations are felt as if addressing people’s epistemic needs. As such, we can interpret ruminating as a kind of epistemic practice. Furthermore, in lived experience, ruminating appears as a specific way of being-in-the-world. It is an integrated experience, combining negative emotions, unpleasant bodily feelings, and maladaptive self-narratives. Ruminating is temporally extended, bridging past, present, and future, drawing on core memories to support maladaptive conclusions. Finally, ruminating is an environmentally situated experience, with certain contexts (e.g., smoking, going for a walk, scrolling on social media) being more conducive to ruminating.

We did not observe meaningful experiential differences in the phenomenological structure of ruminating between the normative and clinical samples. This is consistent with the idea that ruminating can occur subclinically in the normative population as well (134), and that it is a transdiagnostic construct that spans ordinary overthinking to pathological hyperreflexivity (1).

Our qualitative material revealed that ruminating may amount to an epistemic (i.e., knowledge-forming) practice. As such, it is closely related to epistemic needs; that is, the need to know (135). The relationship between ruminating and knowledge has long been established in the literature. People who have a tendency to ruminate also exhibit low tolerance for uncertainty (136). Furthermore, we observed ruminating to be associated with a sense of satisfaction, of figuring something out, which is consistent with earlier findings that show ruminating to be essentially a (meta)cognitive coping mechanism (137). Ruminations are characterized by a problematic epistemic status, wherein the events that are being pondered about are typically skewed or the consequences of one’s perceived failures are exaggerated. This observation maps well onto the notion of catastrophizing, which is closely linked with rumination-proneness (138). However, earlier work typically conceptualizes ruminating either as a specific style of responding to negative events, a cognitive bias, or a maladaptive emotion regulation strategy (8, 62, 137). Our findings suggest that the knowledge-forming component of ruminating is not merely a maladaptive belief (i.e., what people believe ruminating leads to) but its structural component (i.e., what ruminating is).

In phenomenological literature, the *natural attitude* denotes a set of prereflective assumptions that allow us a pragmatic orientation in our social world (103, 104). As pointed out by Stanghellini (105), when this commonsense understanding of the world breaks down, we are forced into a hyper-reflexive stance. Hyper-reflexivity has typically been investigated within the domain of psychosis spectrum disorders. Rather than unthinkingly living through life, patients start to contemplate every aspect of it, thereby experiencing themselves as abstract, intellectualized, and alienated entities. Our findings suggest that this can be extended to non-psychotic ruminations as well. Experientially, this intellectualization of daily life does not amount only to a cognitive processing style as has been previously suggested (3), but to a whole way of being-in-the-world, a specific assemblage of a person’s lifeworld (107).

This is seen particularly in the observation that under the conditions of a collapsed commonsense understanding of the world, our participants did not ruminate only mentally. They set up external structures (e.g., keeping a journal, getting validation from ChatGPT, going for a walk on the same path as with a former partner) that reinforced ruminating. In other words, ruminating can be “extended” or “scaffolded” (31, 139). Extended cognition is a paradigm that does not assume that cognition amounts merely to an abstract processing of external stimuli but is embodied, situated, and environmentally extended (140). Aspects of our environment (e.g., tools) are not only external factors for our cognition to process but can in and of themselves constitute cognition (141). While extended factors have typically been conceptualized as adaptive scaffoldings (142), aiding in emotion regulation, little work has been done on their maladaptive potential (143). Our findings suggest that people who ruminate construct environments that are conducive to ruminating. Further psychotherapeutically-oriented research is required to shed further light on these dynamics.

Self-narratives generated by ruminating are simplistic, reducing the self to a single qualifier. Such biases are well-reported in the literature. “Motive of the lie” is a phenomenon wherein patients feel that once they have entered a depressive episode, their way of being prior to the illness was inauthentic (77). Similarly, Fedoroff (122) reports that many patients who admit themselves into paraphilia clinics as pedophiles, in fact exhibit no such tendencies. They are depressed and feel like “the worst type of person.” Since pedophiles are commonly perceived as the worst kind of criminals, they assign this label to themselves. Finally, in people with melancholia, self-identity and role identity collapse into a single narrative self (123). Our study suggests that this process is phenomenologically grounded in ruminating wherein people interpret themselves in a simplistic manner.

Our participants described ruminations as embodied. The phenomenological tradition has long discussed one’s bodily attunement to the world (113, 144–146). These feelings are often disturbed in depression. Patients’ bodies are no longer experienced as skillful mediums through which the world is disclosed to them, but become cumbersome and awkward (146). This leads to difficulties with entering into a shared social space, prompting feelings of estrangement and alienation (147, 148). This sense of alienation is reflected in our participants’ tendency for self-objectifying, where their whole life becomes an object of manipulation, typically with the goal of self-improvement. Such “technologies of the self” may be problematic as they can lead to the deepening of symptoms in patients with unstable sense of selfhood (149) and can preclude the reliance on more adaptive strategies of emotion regulation (e.g., intersubjective regulation) (150).

In addition to one’s environment, our participants reported a gap in sensemaking associated with their bodies. Emotions are typically grounded in bodily feelings (151). Patients being unable to assign meaning to bodily experience may provide a phenomenological understanding for the link between rumination and alexithymia (152, 153). Finally, ruminations were observed to be located in specific body parts, a phenomenon that has otherwise been reported in self-disorders under the term spatialization of thoughts (154).

Our data further showed that ruminating exhibits a polymorphic structure. It integrates past, present, and perceived future experience, which are all linked through a bodily resonance. Nolen-Hoeksema (155) has termed this phenomenon the “yeast effect,” wherein a negatively valenced event triggers wide-ranging associations that seem to grow in a person’s awareness. In our participants, single events or concerns tended to expand into complex experiential constellations, confirming this mosaic, temporally extended form.

Our findings are consistent with the *flow* model of ruminating, where they are conceptualized as integrating intention, memory, affect, and external events (156). This observation suggests that ruminating does not amount to a single maladaptive cognitive process (e.g., heightened self-related cognition). Rather, it is an organizing principle that integrates multiple cognitive processes at the same time.

Finally, we observed that ruminating can act as an affective fulcrum: it is felt as if it can retrigger a depressive episode by reorganizing one’s lifeworld. Ruminating predicting recurrence of depressive episodes is well-described in the literature (157, 158). Ruminating works as a cognitive kindling or catalyst as ever smaller cues can become sufficient to trigger a subsequent episode (159, 160). Our findings demonstrate how this kindling works experientially. The lived mechanism is the gradual collapse of commonsense, moving from hyperreflexive epistemic reexamination to unstable knowledge formation to employing externalized scaffoldings to construction of a self-pathologizing worldview and finally to episode onset. This gradual shift, mediated by a particular cognitive style may open avenues for interventional psychiatry wherein tailor-made interventions (e.g., through psychotherapy or neuromodulation) could be employed to prevent it.

Ruminating is thus a cognitive process that cyclically enacts a pathological lifeworld. Depressive states engender alienation and loss of common sense. Ruminating appears as an adaptive solution, because people who ruminate experienced it to be a productive coping mechanism during their development (3). However, it does not create stable knowledge. This is likely both due to the fact that in depression, cognitive processes are biased towards negatively valenced information, as well as people ruminating about processes that are not accessible to conscious reflection (e.g., one’s personality structure) and need to be addressed in an intersubjective context (e.g., psychotherapy) (109–111).

## 5 Limitations and future directions

Ruminating is typically conceptualized as a passive process (3). However, we observed that ruminating is commonly experienced as active. It may be that the narratives of agency reported in this study are an artifact of the method. Our primary methodological framework was micro-phenomenological interview, which assumes that lived experience is meaningfully related to experiential gestures (84, 86). Future studies that are not biased by such assumptions are required to corroborate these findings.

Furthermore, while insightful for understanding how people experience ruminating, it is unclear how these methods could be integrated into psychotherapeutic work. Further theoretical and empirical work is required to ascertain how these findings could best be integrated into clinical practice. The study followed the principles of qualitative phenomenology, wherein participants are included in the study based on their phenomenological aptitude (161) and their interest in participation (76, 96). As such, we cannot exclude self-selection bias. Furthermore, we used mailing list to sample participant from the normative population in which no men volunteered. In Slovenian cultural environment, men are typically reluctant to participate in studies. As such, we cannot state for certainty whether this overrepresentation of women participants in the normative sample is due to the broader cultural factors influencing people’s willingness to participate in scientific studies or generally observed trend for women to ruminate more than men.

The study was cross-sectional in nature. As such, it would be methodologically unsound to extrapolate any specific effect that psychiatric treatment (e.g., psychotherapy, psychopharmacological medication) had on ruminating. Future studies should employ contemporary methods in qualitative phenomenology to investigate changes in ruminative experience before and after treatment. An example of such an approach comes from Medeiros et al. (162) where alterations of ruminative experience were investigated in relation to a mindfulness intervention. In our group, studies are currently underway examining the impact of repeated transcranial magnetic stimulation and s-ketamine on ruminative phenomenology.

Finally, we purposefully chose to not employ established clinical scales for the estimation of ruminating on our participants so as to follow the general principle of qualitative research to reexamine pre-existing assumptions about the nature of a given phenomenon. It would be of interest to the study of ruminating to compare our findings to existing instruments such as the Ruminative Response Scale (132).

## 6 Conclusion

Our study demonstrates how qualitative phenomenology contributes to clinical psychology by revealing experiential structures that remain invisible to symptom-based approaches.

By grounding analysis in participants’ lived experience, we show how ruminating reorganizes embodiment, temporality, affectivity, and worldview. In doing so, the study contributes to bridging phenomenological psychopathology with clinical models of rumination, providing a richer foundation for future empirical and therapeutic developments.

## Data Availability

The original contributions presented in the study are publicly available. This data can be found here: https://osf.io/3xnmu.

## Footnotes

1 Jaspers (113) describes two forms of knowledge: *explanation* [Ger. *Erklärung*] and *understanding* [Ger. *Verstandnis*]. The former refers to the causal connections established by the natural sciences, whereas the latter refers to the tacit, affective, and self-reflective knowledge provided by the humanities. As such, while this subchapter may imply causal statements on our part, this is merely an artefact of language. We are engaging in what is referred to as *genetic analysis*; that is, we are describing a typical sequence of experience as appraised by individual participants, rather than a cause-and-effect dynamic.
